# Health Information on the Web and Consumers’ Perspectives on Health Professionals’ Responses to Information Exchange

**DOI:** 10.2196/med20.3213

**Published:** 2014-07-08

**Authors:** Gül Seçkin

**Affiliations:** ^1^University of North TexasDepartment of SociologyUniversity of North TexasDenton, TXUnited States

**Keywords:** health communication, Internet, information, patient-physician relationship

## Abstract

**Background:**

Health information technology, which is sometimes referred to as informaticization of medicine, is changing the extent to which patients become competent producers of their own health by enabling them access to health information anytime and anywhere.

**Objective:**

This research provides preliminary information on users' perceptions of the extent to which use of the Internet for health information impacts medical encounters. We specifically explored the following questions: (1) To what extent perceptions of positive or negative changes in medical encounters are associated with sociodemographic background of online health information seekers, and how often the Internet information is discussed with providers? (2) To what extent is there an association between perceived changes in medical encounters and frequency of referring to the Internet during medical encounters? (3) To what extent is there an association between sociodemographic background of online health information users and frequency of discussing of the Internet information with providers?

**Methods:**

The data for this study was derived from a national sampling of online health and medical information users who participated in the Study of Health and Medical Information in Cyberspace—Survey of User Perceptions (N=710). This study used a nationally representative online research panel of the US adults maintained by the Knowledge Networks. Analysis of variance (ANOVA), chi-square, and *t* tests were performed to examine the data.

**Results:**

Although Internet sources allow people the opportunity to gather health or medical information, discussion of this information was not a very common activity. It is noteworthy that half of the sample never or rarely discussed health/medical information obtained from Internet sources with health professionals. Chi-square analyses revealed that discussion of online health information with providers were associated with education, income, and marital status. We also found that discussion of the Internet information mostly promotes better physician-patient interactions. Analyses with post-hoc tests identified that perceived changes in medical encounters were associated with age, education, and income. However, 9.1% (64/703) of our respondents strongly agreed that the interactions with their providers have been strained. *T* test analyses showed that marital status, race, and gender were not significant.

**Conclusions:**

Embracing new technologies, and adapting to changing roles and relationships in delivery of medical care are critical to effective delivery of patient-centered care. Health professionals could also guide patients on how to evaluate information and where to access to reliable and accurate information.

## Introduction

Evidence from nationally representative surveys show that nearly half of the US population have sought health-related information on the Internet [1]. A 2010 presidential commission report underscored the importance of health information technology in enabling every consumer access to information they need [1]. Technological reinvention of the way information is created, distributed, and retrieved has led to a thriving movement within the health care system and the medical culture. In fact, the Internet has become a platform for health care information and support to the extent that more than 110 million Americans obtain their health information from Web-based sources [2]. As more patients retrieve health and medical information when and where they need it, they also desire a more active role in their own care and clinical decision making. Previous literature commonly reported that the power dynamics in medical encounters require patients to become well-informed if they prefer to take a proactive approach and be treated with due respect as health care partners [3]. Accordingly, today, in the age of post-information society, it is a common practice that health consumers turn to the Internet first before visiting a physician [4]. Sometimes referred to as the informaticization of medicine, cyber patients surf the virtual library of health and medical information to equip themselves with competencies as they navigate themselves through medical system in offline world [4]. The users of online information access medical knowledge outside of the venue of consulting rooms and they decide on the content and amount of information received. Accordingly, it has been suggested that the Internet provides patients with opportunities to display the modern marker of being a responsible, proactive, and competent patient in the age of information and communication technologies [5].

However, little is known about how obtaining health or medical information from Web-based sources impacts the provider-patient relationship [6]. The Internet is challenging the traditional hierarchical patterns of information provision that used to exist in medical encounters. As patients use the Internet to gather information, the patient-provider dynamic may change in various ways. [7]. Existing reports about the implications of the Internet technology for health care services are conflicting. The Internet is argued to transform the physician-patient interaction by demystifying medical expertise and by redefining patients as eHealth information managers [8]. While some providers may welcome the opportunity to collaborate with proactively informed patients, other providers may feel challenged or their expertise being questioned [9]. A study of oncologists found that health professionals perceive availability of digital health information sources as a positive development, while some embrace this less [10]. Some research found that clinicians react negatively and feel challenged when patients bring information retrieved from the Internet to medical consultations [4]. This is especially the case in instances where Web-based information does not coincide with medical facts and professional opinions [11]. Research also shows that most Internet users do not discuss the Web-based information with their clinicians due to hesitation to over-step the boundary between physician-patient interactions, and concerns about alienating their physicians by making them feel not trusted [12]. The hierarchical interaction between patients and physicians may diminish the tendency of patients to reveal that they were looking up information from alternative sources, which, in turn may lead to concerns about jeopardizing the quality of health and medical information received.

The current study examined the extent to which gathering health or medical information from the Web resources is perceived as effective medical interactions. This study also examined to what extent perceptions of change in health care interactions are associated with how often Internet information is discussed, and to what extent do people feel that interactions with their health care providers are strained as a result of referring to the information obtained from the Web resources. The impact of patients’ sociodemographic backgrounds on these perceptions are also reported.

## Methods

### Data Source and Ethical Approval

The current study used a nationally representative online research panel of US adults maintained by the Knowledge Networks. Knowledge Networks is a non-profit and academic research firm that has recruited the first online research panel representative of the US population. Data was obtained from national sampling of online health and medical information users who participated in the Study of Health and Medical Information in Cyberspace- Survey of User Perceptions. The survey, which consisted of 50 questions, was self-administered and accessible for a designated period of time. Participants were able to complete the survey only once. Knowledge Networks contacted approximately 1000 panelists, of which 710 completed the survey. The inclusion criteria in this study were using the Internet, at least occasionally, to look for health-related information and being at least 18 years of age.

The survey asked the respondents to report whether and to what extent they (1) utilize the Internet to obtain health related information, 2) evaluate the credibility and quality of the Internet information, (3) take action to manage their health based on the Internet obtained information, and (4) perceive encounters with providers are affected by seeking health or medical information on the Internet themselves. Each item was measured on a 5-point Likert scale ranging from 1, never/strongly disagree/not at all to 5, always/strongly agree/very much. Before launching the survey, the items were first pilot tested (n=10). Ethics approval was obtained during the recruitment process before the respondents joined the Knowledge Networks panel. Approval of the Institutional Review Board of the University of Maryland, Baltimore County was also obtained.

### Measures

Demographic and socioeconomic covariates included race/ethnicity, education, income, gender, age, race, and marital status. Age was grouped into four groups: (1) 18-29, (2) 30-44, (3) 45-59, and (4) 60 and older. Gender was coded as (0) male and (1) female. Response categories for race/ethnicity, and marital status were collapsed to account for small cell sizes and were measured as dichotomous variables. Race/ethnicity was measured as (0) Caucasian and (1) minority. Marital status was measured as (0) nonmarried/nonpartnered and (1) married/ partnered. Education was coded as (1) high school or less, (2) some college or associate degree, (3) college degree, and (4) post-graduate degree. Annual family income was categorized into four groups: (1) $29,999 or less, (2) $30,000-$59,999, (3) $60,000-$99,999, and (4) $100,000 and above.

Frequency of seeking health or medical information from the Internet was assessed with a single question: “How often do you seek health or medical information on the Internet?” The response options ranged from (1) never to (5) always. Frequency of discussing online information with health providers was also assessed with a single question: “How often do you discuss the information you obtain from the Internet with a health care provider?” The response options ranged from (1) never to (5) always.

Perceived changes in interactions with health care providers were assessed with 4 items which asked the respondents to indicate the extent to which they agreed with the following statements: (1) ‘’I receive more attention to my questions from health care providers as a result of gathering health or medical information from the Internet,’’ (2) ‘’I receive more information to my satisfaction from health care providers as a result of gathering health or medical information from the Internet,’’ (3) ‘’Interactions of health care providers with me have become more respectful as a result of gathering health or medical information from the Internet,’’ and (4) ‘’interactions with health care providers have become strained as a result of bringing in health or medical information from the Internet to the appointments.’’ The response options ranged from (1) strongly disagree to (5) strongly agree. The last item was reverse coded to be consisted with the other items.

An estimation of the factor structure of these four items using rotated solution with the Varimax method and the Scree plot suggested a two-factor solution. Internal consistency reliability estimate also showed that dropping the last item from the composite scale would increase Cronbach alpha from .59 to .87. Thus, an index score for perceived positive changes in health care interactions was calculated by taking the average of the standardized scores on only the first three items. The minimum score was 1 and the maximum score was 5 with higher scores indicating a greater perceived positive changes. The last item was analyzed separately as a single item of perceived negative change in health care interactions. The response options ranged from (1) strongly disagree to (5) strongly agree with higher scores indicating higher levels of perceived strain.

### Statistical Analysis

Chi-square analysis, *t* test, and analysis of variance (ANOVA) were performed to examine whether significant associations exist between sociodemographic factors, discussion of Web-based information with health providers, and perceived impact of gathering information from Web resources on medical encounters. Data were analyzed using IBM SPSS Statistics version 21.

## Results

The survey sample included adults who ranged in age from 18 to 93 with a mean of 48.82. There were 15.2% (108/710) of respondents younger than 29 years of age and 27.3% (194/710) older than 60 years of age. Just over half of the sample were women (53.7%, 381/710). The majority of the sample was Caucasian (76.5%, 543/710) and married or partnered (67.8%, 481/710). College graduates and those with post-graduate degrees comprised 37.3% (265/710) of the sample while a similar percentage of the sample had high school or less education (33.4%, 237/710). Over half of the sample (57.0%, 405/710) reported an annual household income of at least $60,000 USD. We found that while 12.4% (88/710) reported frequently seeking health related information on the Internet, one-third reported rarely (32.4%, 230/710). Half of the sample indicated never or rarely discussing the Internet information with health providers (50.0%, 351/702) while 12.6% (89/702) reported frequently. The correlation between frequency of seeking health/medical information on the Internet and frequency of discussing it with providers is significant (*r*=.33, *P*<.001). Chi-square analyses revealed a few significant differences associated with discussion of Internet-based information with health care providers. Higher percentages of individuals with college or more education (χ²=26.78, *P*=.001), those from upper income brackets (χ²=13.97, *P*=.001), and those who were married/partnered individuals (χ²=21.80, *P*=.001) engaged in more frequent discussions of Internet information. Approximately half of those who frequently discussed the Internet information with providers had a college degree or more (47%, 42/89) compared to one-fifth (20%, 18/89) who reported a high school diploma or less. Regarding income, respondents who discussed the Internet information frequently, only 6% (5/89) earned less than $30,000 USD while 67% (60/89) reported earning $60,000 USD or more. Chi-square analyses revealed that age, race/ethnicity, gender, and marital status were not significantly associated with discussing the Internet information.

As for perceived changes in medical encounters, one–third (31.3%, 220/702) strongly agreed to the item “I receive more attention to my questions” and nearly over one-third (35.9%, 253/704) indicated strong agreement to “I receive more information to my satisfaction”. In response to the statement “interactions of health care providers have become more respectful”, while 16.4% (115/702) reported a strong agreement, nearly one-quarter (23.8%, 167/702) reported disagreement indicating no such positive change. On the item measuring perceived strain, 9.1% (64/703) reported strong agreement, while almost half of the sample (48.8%, 343/702) reported disagreement. The composite scale of perceived positive changes showed an overwhelming majority (74.1%, 526/710) reported positive changes ranging from somewhat agreeing (61.8%, 439/710) to strongly agreeing (12.3%, 87/710). However, 25.9% (184/710) reported no such changes in health care interactions. Chi-square tests also identified several variables that were associated with frequency of discussing information obtained from the Internet with perceived changes in health care interactions. Nearly 63% (56/89) of those who frequently discussed information with professionals reported strong agreement to the item “receiving more attention to their questions from health providers” while the percentage for those who engaged in rare discussions of online information was 17.6% (61/346). Similarly, while 63% (56/89) of those who engaged in frequent discussions strongly agreed that they received more information to their satisfaction. However, the percentage was 21.0% (73/348) for those who rarely engaged in such discussions. A higher percentage of people who reported frequent discussions also strongly agreed that there was an increased respect in health care interactions (35%, 31/89) compared to those who did not (8.6%, 30/347). Comparison of discussers to non-discussers showed that nearly 34% (30/89) of frequent discussers agreed strongly to positive changes on the summated scale compared to nearly 5% (19/348) of non-discussers. Lastly, almost 9% (30/348) of the respondents who rarely discussed Web information reported strong agreement to strained health care interactions. In contrast, nearly twice that percentage was reported by those who frequently broached up the topic of information obtained from the Internet (17%, 15/88). Detailed percentages, chi-square values, and corresponding significance levels are shown in Table 1.

ANOVA analyses with post-hoc tests also identified several sociodemographic variables that were significantly associated with perceived changes in medical encounters. These factors are age, education, and income. Respondents older than 60 years of age reported less perceived strain in medical encounters than those between the ages of 18-29 (mean 2.40 vs mean 2.66, *P*=.033). Respondents with “some college or less” education perceived an increase in respect as a result of gathering information from the Internet (M=3.05 vs M=2.81, *P*=.010). There is also a marginally significant association between higher income and receiving more information to satisfaction (mean 3.26 vs mean 3.03, *P*=.058). *T* test analyses showed that marital status, race, and gender were not significant correlates of perceived changes in medical encounters. ANOVA and *t* test results are shown in Table 2 below.

**Table 1:** Covariates stratified by discussing Internet information with health care providers.

Covariates		Full sample characteristics	Never/Rarely	Sometimes	Mostly/Always	χ^2^ (df)	*P*
		n (%)	n (%)	n (%)	n (%)		
**Age, mean (SD)**		48.82 (16.43)	49.98 (10.91)	48.88 (15.65)	46.97 (15.19)	5.637 (6)	.465
	18-29	100 (15.2)	57 (16.2)	39 (14.9)	11 (12.4)	
	30-44	175 (24.6)	86 (24.5)	59 (22.5)	28 (31.5)	
	45-59	233 (32.8)	106 (30.2)	96 (36.6)	28 (31.5)	
	60 and older	194 (27.3)	102 (29.1)	68 (26.0)	22 (24.7)		
**Gender**						5.115 (2)	.077
	Female	381 (53.7)	176 (50.1)	145 (55.3)	56 (62.9)	
	Male	329 (46.3)	175 (49.9)	117 (44.7)	33 (37.1)	
**Education, mean (SD)**		2.97 (0.96)	2.79 (0.97)	3.12 (0.93)	3.24 (0.85)	26.943 (6)	.00
	High school or less	237 (33.4)	143 (40.7)	72 (27.5)	18 (20.2)	
	Some college	208 (29.3)	105 (29.9)	72 (27.5)	29 (32.6)	
	College degree	153 (21.5)	61 (17.4)	69 (26.3)	22 (24.7)	
	Post graduate degree	112 (15.8)	42 (12.0)	49 (18.7)	20 (22.5)	
**Income, mean (SD)**		2.70 (1.07)	2.60 (1.11)	2.75 (1.07)	2.94 (0.90)	13.974 (6)	.030
	$29,999 or less	122 (17.2)	74 (21.1)	41 (15.6)	5 (5.6)	
	$30,000-$ 59,999	183 (25.8)	90 (25.6)	67 (25.6)	24 (27.0)	
	$60,000-$ 99,999	191 (26.9)	87 (24.8)	70 (26.7)	31 (34.8)	
	$100,000 or more	214 (30.1)	100 (28.5)	84 (32.1)	29 (32.6)	
**Marital status**						21.80 (10)	.001
	Married	481 (67.8)	208 (59.3)	161 (61.5)	53 (59.6)		
	Non-married	229 (32.3)	143 (40.7)	101 (38.5)	36 (40.4)		
**Race/Ethnicity**						1.126 (2)	.569
	White	543 (76.5)	265 (75.5)	206 (78.6)	66 (74.2)	
	Non-White	167 (23.6)	86 (24.5)	56 (21.4)	23 (25.8)	
**Receiving more attention to questions, mean (SD)**		3.12 (0.83)	2.86 (0.79)	3.28 (0.72)	3.64 (0.89)	90.041 (4)	.001
	Disagree	132 (18.8)	94 (27.2)	28 (10.7)	9 (10.1)	
	Somewhat agree	350 (49.9)	191 (55.2)	134 (51.3)	24 (27.0)	
	Agree	220 (31.3)	61 (17.6)	99 (37.9)	56 (62.9)	
**Receive more information, mean (SD)**		3.21 (0.81)	2.93 (0.78)	3.41 (0.67)	3.66 (0.85)	90.384 (4)	.001
	Disagree	109 (15.5)	83 (23.9)	17 (6.5)	8 (9.0)	
	Somewhat agree	342 (48.6)	192 (55.2)	123 (47.1)	25 (28.1)	
	Agree	253 (35.9)	73 (21.0)	121 (46.4)	56 (62.9)	
**Receive more respect, mean (SD)**		2.91 (0.78)	2.73 (0.72)	3.04 (0.68)	3.28 (0.82)	54.651 (4)	.001
	Disagree	167 (23.8)	111 (32.0)	43 (16.5)	12 (13.5)	
	Somewhat agree	420 (59.8)	206 (59.4)	164 (63.1)	46 (51.7)	
	Agree	115 (16.4)	30 (8.6)	53 (20.4)	31 (34.8)	
**Positive changes in health care interactions, mean (SD)**		3.08 (0.71)	1.68 (0.57)	3.24 (0.58)	3.53(0.76)	86.924 (4)	.001
	Disagree	184 (25.9)	130 (37.4)	42 (16.0)	10 (11.2)	
	Somewhat agree	439 (61.8)	199 (57.2)	184 (70.2)	49 (55.1)	
	Agree	87 (12.3)	19 (5.5)	36 (13.7)	30 (33.7)	
**Interactions strained, mean (SD)**		2.50 (0.82)	2.55 (0.80)	2.45 (0.75)	2.49 (1.03)	19.034 (4)	.001
	Disagree	343 (48.8)	152 (43.7)	139 (53.5)	49 (55.7)	
	Somewhat agree	296 (42.1)	166 (47.7)	103 (39.6)	24 (27.3)	
	Agree	64 (9.1)	30 (8.6)	18 (6.9)	15 (17.0)	

**Table 2:** Covariates stratified by perceived impact of discussing health/medical information from the Internet on medical encounters.

Covariates		More attention	More information	More respect	Positive changes in health care		Interactions strained
		Mean (SD)	Mean (SD)	Mean (SD)	Mean (SD)		Mean (SD)
**Age**							
	18-29	3.03 (0.93)	3.08 (0.96)	2.86 (0.89)	2.91 (0.77)		2.66 (0.96)
	30-44	3.13 (0.79)	3.22 (0.73)	2.92 (0.73)	2.96 (0.56)		2.57 (0.81)
	45-59	3.14 (0.80)	3.21 (0.76)	2.92 (0.72)	2.94 (0.57)		2.47 (0.77)
	60 and older	3.13 (0.83)	3.26 (0.83)	2.93 (0.71)	2.92 (0.56)		2.40 (0.77)
	*F* test	*F*_3,701_= 0.509	*F*_3,700_=1.096	*F*_3,701_=0.235	*F*_3,701_=0.202		*F*_3,699_= 2.920
	*P* value	.676	.350	.872	.895		.033
**Gender**							
	Female	3.09 (0.86)	3.22 (0.78)	2.89 (0.77)	2.91 (0.62)		2.48 (0.81)
	Male	3.15 (0.80)	3.20 (0.83)	2.94 (0.72)	2.96 (0.58)		2.54 (0.82)
	*t*_df_ test	*t*_700_ = 0.986	*t*_736_ = 0.337	*t*_700_ = 0.864	*t*_703_ = 1.097		*t*_701_ = 1.012
	*P* value	.324	.736	.388	.273		.312
**Race/Ethnicity**							
	White	3.13 (0.81)	3.21 (0.79)	2.90 (0.72)	2.93 (0.58)		2.48 (0.80)
	Non-White	3.09 (0.89)	3.19 (0.86)	2.96 (0.82)	2.96 (0.68)		2.59 (0.86)
	*t*_df_ test	*t*_700_ = 0.471	*t*_702_ = 0.333	*t*_700_ = 0.949	*t*_703_ = 0.570		*t*_701_ = 1.445
	*P* value	.637	.740	.343	.569		.149
**Education**							
	High school or less	3.09 (0.86)	3.14 (0.86)	2.91 (0.77)	2.93 (0.65)		2.56 (0.85)
	Some college	3.17 (0.83)	3.27 (0.80)	3.05 (0.76)	3.01 (0.61)		2.55 (0.81)
	College degree	3.08 (0.77)	3.18 (0.74)	2.81 (0.70)	2.89 (0.55)		2.47 (0.78)
	Post graduate	3.11 (0.86)	3.27 (0.78)	2.82 (0.71)	2.94 (0.55)		2.36 (0.76)
	*F* test	*F*_3,698_=.466	*F*_3,700_=1.154	*F*_3,698_=3.829	*F*_3,701_=1.647		*F*_3,699_=1.921
	*P* value	.706	.326	.010	.177		.125
**Income**							
	$29,999 or less	2.98 (0.85)	3.03 (0.86)	2.93 (0.80)	2.85 (0.64)		2.46 (0.82)
	$30,000-$59,999	3.15 (0.84)	3.23 (0.82)	2.93 (0.77)	2.97 (0.61)		2.58 (0.80)
	$60,000-$99,999	3.12 (0.81)	3.26 (0.77)	2.89 (0.75)	2.94 (0.57)		2.50 (0.83)
	$100,000 or above	3.17 (0.83)	3.25 (0.78)	2.92 (0.70)	2.95 (0.59)		2.47 (0.81)
	*F* test	*F*_3,698_ = 1.582	*F*_3,700_=2.509	*F*_3,698_=0.126	*F*_3,701_=1.186		*F*_3,699_ 0.851
	*P* value	.192	.058	.945	.314		.466
**Marital status**							
	Married	3.13 (0.79)	3.23 (0.75)	2.89 (0.71)	2.93 (0.55)		2.48 (0.78)
	Non-married	3.10 (0.89)	3.18 (0.89)	2.95 (0.80)	2.94 (0.67)		2.54 (0.86)
	*t*_df_ test	*t*_700_ = 0.392	*t*_702_ = 0.836	*t*_700_ = 0.914	*t*_701_ = 0.181		*t*_701_ = 0.814
	*P* value	.702	.404	.361	.856		.416

## Discussion

### Principal Findings

This study examined sociodemographic correlates of discussing Internet-based information with health care providers. We also examined whether respondents’ reports of perceived changes in medical encounters were associated with their sociodemographic background characteristics and how often they engaged in discussion of information from Web sources.

First, a striking finding from this research is that an overwhelming majority (87.6%, 622/710) reported sometimes or rarely searching for health or medical information on the Internet. This might be because most participants were healthy and did not feel the need to search for such information. In fact, a survey by the Pew Research Center’s Internet & American Life Project showed 80% of American adults reported their health as excellent or good and do not frequently access health information [13]. Alternatively, as data from the Health Information National Trends Survey (HINTS) indicate that despite wide availability of online health or medical information, the public trust in Internet-based information has decreased and the majority of Americans prefer health care professionals as more trusted source of information [14]. This interpretation is also consistent with the Pew research results that nearly 90% of all adults turn to a health professional when they need information or assistance in dealing with health or medical issue. The Pew report states ‘’American adults continue to turn to traditional sources of health information, even as many of them deepen their engagement with the online world’’ [15].

In corroboration of the HINTS results, this study also revealed differences in use of the Internet health information by sociodemographic characteristics. Specifically, use of the Internet for health purposes was found to be more common among females, Caucasians, and younger people. The fact that half of the respondents were women is consistent with other studies showing closing gender gap in usage of the Internet technology [2]. However, the fact that the majority of the respondents were Caucasian and in higher income brackets is suggestive of persistent digital divide based on racial/ethnic and economic inequalities. Nearly one-third of participants who reported searching the Internet for health information were older adults. This might be indicative of growing popularity of the Internet among cohorts of older adults and narrowing of the generational digital divide.

It is noteworthy that half of the sample never or rarely discussed health/medical information obtained from the Internet with their health care providers. Some of the sociodemographic variables, such as gender and race/ethnicity, that are traditionally associated with online health-seeking behavior were not found to be significantly associated with frequency of discussing Web information with health professionals. Similar to the results of this survey, the Pew survey showed that over 60% of people who look up health information on the Internet reported never or sometimes discussing information they found on the Internet with health care professionals [13]. This could be due to structural limitations of the health care system limiting time that could be set aside for discussion of information from outside sources, or because of patient concerns for not to be perceived as challenging the authority and expertise of their care providers [16]. Although Internet sources allow people the opportunity to gather health or medical information, discussion of it was not found to be a very common activity.

One caveat noted in the literature is that those with poor health or sicker patients were more likely to talk with a clinician about what they found on the Internet [17]. The Pew Research Center’s Internet & American Life Project reported that those who reported worse health status and people living with chronic conditions used Internet health information more frequently when they have access to the Internet and also more frequently discussed Internet information with health care providers [13]. Another research also reported that less than one-third of people who indicated a good health status discussed the Internet health information with their health care provider [16,17].

The results suggest that higher education and income seem to be enabling factors for engagement in discussion of online health information with providers as they were found to be significant in chi-square analyses. This is mostly due to probability that people with higher education and income use the Internet more often, which is also consistent with previous research that health information seeking was more common among higher education and income groups [18,19]. Being in a relationship with a significant other also seems to be another enabling factor. In fact, research has shown that having a partner encourages one to become more assertive and proactive in asking questions to a provider during medical appointments [20].

Regarding the impact of bringing in information from Web sources to appointments and discussing it with providers, we found that it mostly promotes better medical encounters rather than straining it. An overwhelming majority perceived their questions resulted in more attention from their providers and more information provided to their satisfaction. Sense of being more respected as a health care partner is also reported. This could be due to health providers’ appreciation of their patients’ efforts to become more proactive in maintaining and/or regaining their health, and possibly perceiving their patients as informed partners rather than passive and helpless consumers of their services [21].

### Limitations

We should interpret these results with caution that almost half of the sample reported some strain due to bringing in the Internet information to their appointments. Nine percent of our respondents strongly agreed that the interactions with their providers had been strained. It is noteworthy that older adults reported less strain compared to adults in 18-29 age group. This might be related to health providers’ appreciation of use of the Internet by older adults, an age group less expected to use technology in an effort to be in charge of their health and well-being. In contrast, younger adults bringing in information obtained from the Internet might be perceived as challenging the “informational” authority and expertise of health care providers with their technological gadgets or toys. This is an interesting area of further inquiry in order to better understand the age or cohort factor that our results suggested. Another interesting result out of this study is reports of perceived increase in respect by those with “some college” education. This could possibly be due to feeling more confident in interacting with health professionals as a result of gathering information or a real change in attitudes of health providers in interacting with patients who might be less expected to gather information in order to discuss it during medical appointments.

Even though inquiring into health status of the study participants in the current study would have enabled us to analyze the synergy between self-reported health status, frequency of using the Internet for health information, and discussion of it with providers in-depth, the funding limitations constrained the number of questions that could be explored in the survey. Another area of limitation of the current study is that those with chronic health issues or serious diseases may use the Internet in more targeted ways than those who browse the Internet for general health purposes, which in turn, may affect medical encounters differently and provoke differential reactions from providers. The Pew Internet Health Tracking Survey results indicate that the diagnosis of a chronic condition makes a difference in the extent to which people with serious health concerns conduct targeted and specific online research [13,15]. The HINTS also found that there are differences in use of the Internet for health purposes by those who are more sick or have a serious disease compared to those who reported no conditions or being healthier. Even though those who are in poor health may be less likely to be online, they tend to gather more in-depth information when online more frequently [13]. Other research also reported that those with serious chronic illnesses consult the Internet resources for specific information, such as on their doctors’ expertise, a certain medical treatment, or medications [17].

Among the other limitations of the current study is that we could not explore health insurance status, and rate of use of health care services by the study participants. Moreover, the survey could not inquire about the type of Internet sites the respondents were visiting. Future research that would directly observe how patient-provider interactions are affected by patients’ use of the Internet health information resources will help us better understand the various dynamics involved. We also need to understand whether online information results in patient requests such as for additional tests or procedures. Due to a limited set of questions used in the current survey, we were also unable to probe into potential causes of perceived strain and perceived changes in medical encounters. Additional research is also needed to examine whether and how information obtained from the Web sources is integrated into self-care.

### Future Studies

Future papers out of our survey data will analyze questions that inquired about patient non-adherence and non-compliance as a result of using the Internet health or medical information, trust in Internet provided health or medical information, and self-reported ability to evaluate quality and credibility of the Internet health or medical information.

### Conclusions

The Internet empowers patients with broader and richer sources of information if there is a timely and satisfactory health information exchange [22]. In today’s complicated health care context, patients explore their options in order to participate in management of their care [23]. They desire up-to-date information to improve the quality and efficiency of services they receive [24]. Embracing new technologies, and adapting to changing roles and relationships in delivery of medical care are critical to effective delivery of patient-centered care [1]. Health professionals could also help patients get quality health information by guiding patients on how to evaluate information and where to access reliable and accurate information online [25].
